# Genetically Reprogramming Crops and Rhizobacteria for Nutritional Iron Biofortification

**DOI:** 10.1021/acssynbio.5c00614

**Published:** 2025-11-18

**Authors:** Taden B. Welsh, Christopher M. Dundas

**Affiliations:** 1 Department of Plant Biology, 1355 University of Georgia, Athens, Georgia 30602, United States; 2 Institute of Bioinformatics, University of Georgia, Athens, Georgia 30602, United States

## Abstract

Iron is a critical micronutrient for both plants and humans, yet its declining availability across agricultural systems threatens global food security and health. Biofortification of food crops has emerged as a promising strategy to combat iron deficiency and anemia, leveraging both crop breeding and microbiome-based approaches to enhance iron mobilization and uptake. Advances in plant and bacterial synthetic biology could enable the precise programming of iron homeostasis and acquisition mechanisms, offering tailored solutions across diverse species and environments. Here, we outline key biomolecules, genes, and biosynthetic and transport pathways that represent underexplored synthetic biology targets for improving crop iron acquisition. We highlight opportunities to tune expression strength, tissue specificity, and cross-host pathway transfer to enhance chelation- and reduction-mediated solubilization of soil iron and augment plant uptake. Finally, we emphasize the broader importance of developing plant–microbe–metal actuators as modular components in genetic circuit design and discuss how their deployment across diverse plant and microbial chassis could accelerate agricultural biofortification and improve global nutrition.

## Graphical abstract

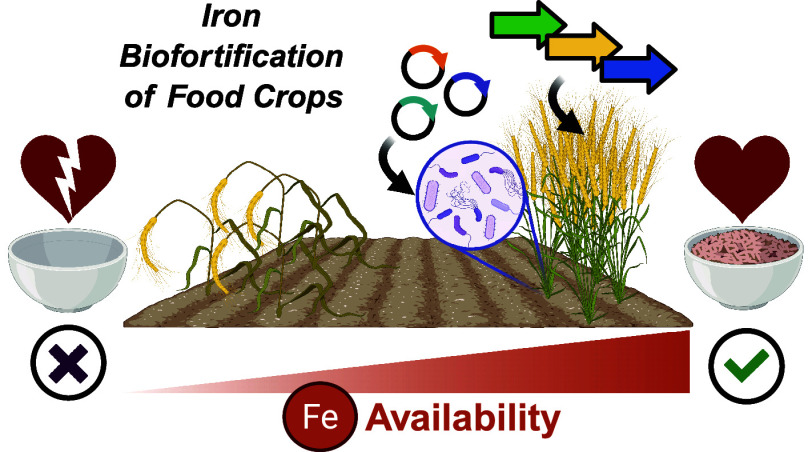

## Challenges and Opportunities for Engineering Iron Homeostasis and Bioavailability in Food

Iron is a critical micronutrient across all kingdoms of life, but its abundance and bioavailability are at risk in both human diets and agricultural soils (Figure 1a). An estimated 25% of the world population is afflicted with anemia, where iron deficiency is a significant contributing factor.^1^ Similarly, plant health is intrinsically tied to adequate iron levels across tissues, and cultivation in iron-inaccessible environments leads to significant physiological deficiencies and decreased crop yields.^2^ Calcareous and other alkaline soils (pH > 6), which cover up to 30% of the world’s land area,^3^ are particularly problematic because, despite containing abundant total iron, they favor the formation of poorly soluble, nonbioavailable Fe^3+^ species like ferric oxide minerals. Under these conditions, plants that cannot effectively acquire iron exhibit reduced photosynthetic activity caused by impaired chlorophyll synthesis and disruption of iron-dependent electron transport through photosystem 1 (PSI), leading to chlorosis, plant growth inhibition, and reproductive issues.^4–6^ A wide range of stressors may further compromise iron bioavailability and uptake in plants, including abiotic factors such as drought and salinity, and biotic stressors like microbial phytopathogens and microbiome dysbiosis.^7,8^ Major crops highly susceptible to iron stress include soybean, citrus, sorghum, and members of the Brassicaceae family.^9^ Although plants deploy native iron acquisition machinery to solubilize and absorb iron from soil, these mechanisms are often inefficient, and deficiency persists. Fertilizer-based supplementation can partially alleviate iron limitation, most commonly through foliar sprays in which iron solutions (typically 1–29 mM Fe) are absorbed directly through the leaf cuticle.^10^ However, adoption of these practices is constrained by agronomic logistics, economic cost, and environmental risks such as runoff.^11^ Because dietary iron ultimately originates from crops and crop-fed animals, developing effective strategies for maintaining high iron levels across agricultural plants is paramount for food security and human health.

**Figure 1:**
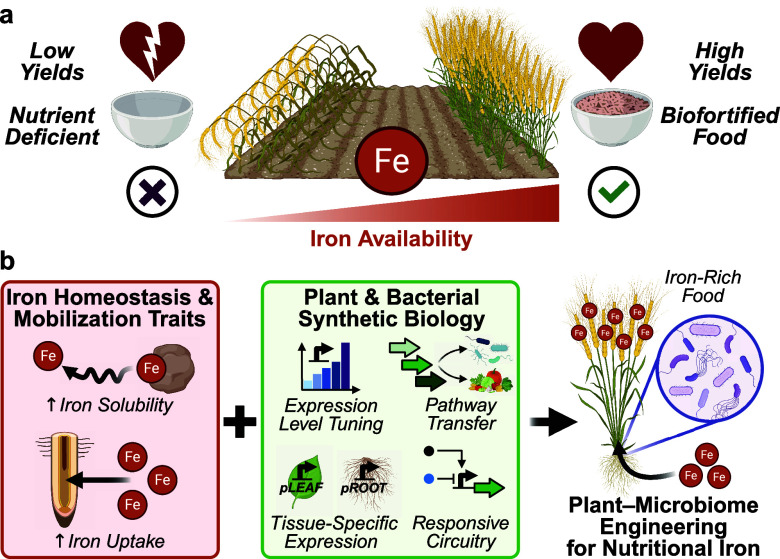
Synthetic biology strategies in plants and bacteria to overcome iron limitation in food crops. (a) Iron availability influences crop yields and nutritional quality. (b) Iron homeostasis and mobilization traits can be engineered through plant and bacterial synthetic biology, enabling the development of integrated plant–microbiome systems for iron biofortification.

Biobased iron fortification offers a sustainable alternative to chemical fertilizers by enabling targeted improvements in iron uptake and sequestration by food crops. For example, the root microbiome plays a key role in regulating plant iron acquisition, with microbial bioinoculants promoting iron solubilization and uptake.^12^ Separately, crop breeding programs have focused on developing elite genotypes possessing allelic improvements that enhance iron accumulation and resilience to iron-related stress.^13–15^ A relatively untapped strategy is to apply synthetic biology, including designer gene circuits and metabolic engineering, to precisely program iron homeostasis and mobilization pathways in both plants and their associated soil microbes (Figure 1b). Genes in these pathways encode key functions, including chelator and reductase biosynthesis, iron solubilization, and transport into plant tissues, and have been predominantly manipulated through simple overexpression or genomic knockouts.^5,6,16^ In contrast, fine-tuned and synthetic control of these pathways through quantitative modulation of gene expression, extension beyond native spatiotemporal patterns (including cell types, tissues, and developmental stages), and heterologous transfer into non-native plant species or microbial hosts remain largely unexplored. Integrating synthetic biology approaches across plant–microbe–iron interactions will allow for the creation of crop systems that are more resilient to deficiency, higher-yielding, and fortified with bioavailable iron.

Here, we outline synthetic biology strategies in root-colonizing bacteria (rhizobacteria) and plants for enhancing iron biofortification in food crops. While optimizing supplemented iron uptake (e.g., engineering improved plant absorption of foliar sprays) will be important for soils with a generally low iron content, we focus this perspective on strategies that are broadly applicable across diverse soil types to enhance the extraction of poorly bioavailable iron. In the following sections, we highlight biomolecules, genes, and pathways that offer promising targets for genetic circuit design.

## Designing Microbial Circuits to Boost Plant Iron Access

Microbial inoculants that enhance plant health, termed plant growth-promoting bacteria (PGPB), are promising candidates for engineering iron biofortification in crops. While rhizosphere bioinoculants have been primarily studied as replacements for nitrogen and phosphorus fertilizers,^17,18^ PGPB can also increase plant iron accumulation, improve metal tolerance, and reduce toxic metal uptake.^19–22^ Across diverse environments, including plant roots and aquatic sediments, bacteria naturally mobilize iron through chelation and reduction mechanisms that fuel assimilatory (e.g., biomass incorporation^23^) or dissimilatory (e.g., anaerobic respiration^24–27^ ) processes. Bacterial metabolites or proteins with reduction potentials lower than those of the Fe^3+^/Fe^2+^ couple can thermodynamically drive Fe^3+^ reduction, generating the more soluble and bioavailable Fe^2+^ species (Table 1). With expanding tools for broad-host-range plasmid deployment^28^ and genome engineering across nonmodel rhizobacteria (e.g., CRAGE^29^ and ICE^30^), these native pathways can now be reprogrammed to improve crop iron nutrition through on-demand mobilization of iron for plant uptake (Figure 2a). Rapid assembly of genetic circuits for bacterial iron homeostasis and mobilization could expedite design–build–test–learn cycles in agricultural biotechnology compared with longer plant engineering approaches. These well-characterized yet underexplored bacterial pathways represent a rich opportunity for implementing relatively simple synthetic biology optimizations in PGPB that could substantially improve both plant iron accumulation and nutritional density.

**Figure 2:**
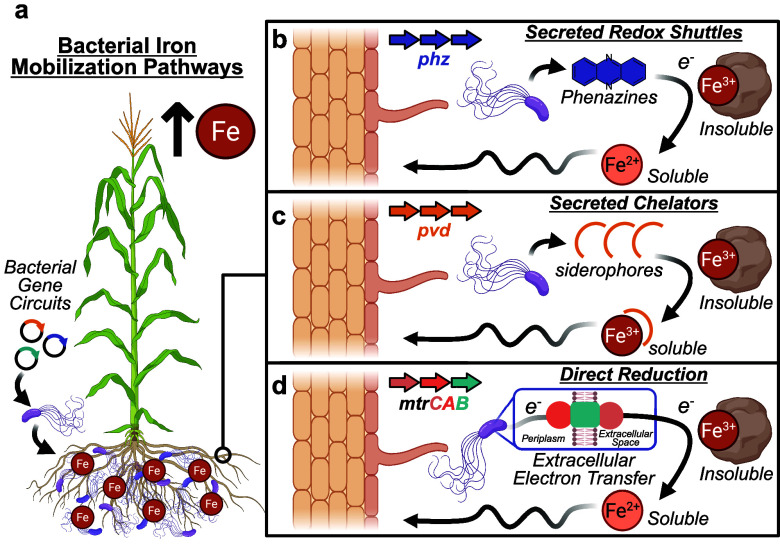
Bacterial targets for enhancing iron availability in the plant microbiome. (a) Engineering plant growth-promoting bacteria (PGPB) with synthetic gene circuits to improve iron biofortification. (b) Secreted redox-active molecules, such as phenazines, solubilize iron for plant uptake. (c) Siderophore secretion chelates insoluble iron, facilitating solubilization and plant uptake. (d) Microbes can increase iron bioavailability to plants by coupling Fe^3+^ reduction to respiration processes.

**Table 1:** Representative Bacterial Engineering Targets for Enhancing Iron Mobilization in the Rhizosphere for Plant Uptake, Highlighting Key Metabolites and Proteins Involved in Fe Solubilization and Reduction

**molecule or protein**	**midpoint potential (mV vs SHE)**	**Fe** ^**3**^^**+**^ **dissociation constant,** ***K***_**d**_ **(M)**	**relevant taxa**	**references**
common redox couples in bacteria				
Fe^3+^/Fe^2+^	+770		universal	
NAD^+^/NADH	–320		universal	^56^
quinone/quinol	–80 to +110		universal	^56^
iron-binding siderophores				
pyoverdine		∼10^–32^	*Pseudomonas*	^57^
pyochelin		∼0.5 × 10^–5^	*Pseudomonas*	^58^,^59^
ferrioxamine B	–450	4 × 10^–30^	*Salmonella, Yersinia*	^60–62^
enterobactin	–750	10^–52^	*Escherichia, Salmonella,*	^63^,^64^
soluble redox shuttles				
pyocyanin	–120		*Pseudomonas*	^65^
phenazine	–252		*Pseudomonas*	^65^
1-hydroxyphenazine	–185		*Pseudomonas*	^65^
phenazine-1-carboxylic acid	–195		*Pseudomonas*	^65^
riboflavin	–208		*Shewanella*	^66^
flavin mononucleotide	∼ −220		*Shewanella*	^66–68^
membrane-bound or extracellular reductases				
MtrC	–250 to 0		*Shewanella*	^69^
OmcZ	–420 to −60		*Geobacter*	^70^

Development of iron biofortification PGPB requires genetically tractable, rhizosphere-adapted chassis strains with innate or engineered capacities for iron mobilization and sequestration. Members of the *Pseudomonas* genus are particularly attractive given their strong root colonization traits^31,32^ and established roles in modulating plant responses to iron stress.^33,34^ For example, *Pseudomonas putida*, *Pseudomonas protegens*, and *Pseudomonas fluorescens* species possess rich genetic toolkits^28,33,35^ and can improve the growth of *Arabidopsis thaliana*, radish, and other crops under iron limitation.^33,34,36^ Less studied *Pseudomonas palmensis* mitigates and rescues the effects of iron–deplete conditions when applied to the rhizosphere of *Nicotiana glauca*.^31^ A compelling molecular target across Pseudomonads is the class of bacterially secreted redox mediators known as phenazines (Figure 2b). Traditionally studied for their antimicrobial properties, growing evidence highlights phenazines as keystone rhizosphere metabolites that facilitate reductive dissolution of insoluble ferrous minerals, thereby promoting mobilization of iron for plant uptake.^32,37,38^ Phenazine biosynthesis pathways, such as the *phz* operon,^39^ could be overexpressed in Pseudomonad bioinoculants to alleviate iron-related crop stress. Furthermore, phenazine production has been shown to improve root colonization of other soil bacteria (e.g., *Dyella japonica* in maize rhizosphere^32^), suggesting that these metabolites may be broadly engineerable across diverse hosts and could enable designer iron fortification bioinoculants based on crop species and soil contexts.

As potent Fe^3+^ solubilizers, siderophores are key targets for microbiome-driven metabolic engineering aimed at enhancing plant iron accessibility (Figure 2c). These secreted compounds exhibit extraordinarily high affinity for Fe^3+^, with dissociation constants as low as ∼10^–51^ M, enabling effective solubilization of soil-bound iron within the rhizosphere for potential uptake by plants.^40^ While essential for bacterial iron uptake, these molecules can also influence plant physiology. Trapet investigated the effects of applying a siderophore-producing *P. fluorescens* to *A. thaliana* roots grown under various iron stresses.^34^ The siderophore produced by *P. fluorescens*, pyoverdine, mitigated iron stress phenotypes in wild-type *A. thaliana*, whereas plant lines with loss-of-function knockouts of an iron transporter (*IRT1*) or iron reductase (*FRO2*) did not exhibit *P. fluorescens*-driven phenotypic rescue. While many siderophores have been identified, optimizing pyoverdine biosynthetic pathways (*pvd*) could be a broadly applicable strategy for engineering iron chelation, given the conservation of these genes across Pseudomonads. *pvd* expression is natively activated when the bioavailable iron concentration is low, enabling increased production of the Fe^3+^ chelator.^41^ Synthetic circuits could drive key pyoverdine biosynthetic genes such as *pvdL* or broader siderophore biosynthesis pathways (Table 1), either constitutively or in response to plant iron stress cues (e.g., rhizosphere acidification).^33,41,42^ However, the impact of these circuits on bioinoculant root colonization and fitness within soil communities must be evaluated to ensure the field persistence of engineered strains.

Electroactive bacteria (EAB) are a class of highly redox-competent microorganisms with extracellular electron transfer capabilities, which could serve as synthetic biology actuators for reductive iron mobilization and plant uptake (Figure 2d). While the abundance and activity of plant-associated EAB across crops remain unclear, their presence in anoxic rhizospheres (e.g., rice^43^ and mangrove^44,45^ ) and the genetic tractability of their Fe^3+^ reduction mechanisms^46^ suggest potential for increasing plant-available Fe^2+^ and serving as iron-biofortifying PGPB. Model EAB, such as *Shewanella* and *Geobacter* species, reduce and solubilize exogenous Fe^3+^ via networks of multiheme cytochromes that span bacterial membranes or form extracellular nanowires.^47^ Although their colonization capacity on food crops is not characterized, experimental evolution could be applied to adapt these EAB for enhanced rhizosphere colonization, likely improving the efficacy of iron mobilization for root uptake.^44^ An alternative strategy is to port EAB iron reduction pathways, including MtrCAB of *Shewanella* and Omc of *Geobacter*, into canonical PGPB chassis that already exhibits high rhizosphere abundance and persistence.^48,49^ The MtrCAB pathway from *Shewanella oneidensis* has been shown to function in *Escherichia coli* and *Marinobacter atlanticus*, which supports its potential transfer into other Gram-negative PGPB species.^48,50,51^ Since EAB iron reduction depends on electron donation from metabolized carbon sources, and not all carbon sources support Fe^3+^ reduction,^52–54^ engineering strains to catabolize prominent root exudates may be necessary to optimize PGPB activity according to their genomic background.^39,55^

## Engineering Plants for Improved Iron Uptake and Sequestration

Plants can be directly engineered to improve iron homeostasis and biofortification traits by targeting native iron acquisition pathways, conventionally designated strategies I or II (Figure 3a and Table 2). Strategy I, used mainly by eudicots, involves acidifying the rhizosphere to increase iron solubility, reducing Fe^3+^ to Fe^2+^ via direct or indirect mechanisms, and transporting highly soluble Fe^2+^ ions into root cells across epidermal membranes.^71^ Strategy II, employed primarily by monocots (grasses), relies on root biosynthesis and exudation of phytosiderophores, which diffuse through soil, chelate poorly soluble Fe^3+^, and are subsequently reabsorbed as iron-chelator complexes.^72^ The genes and regulatory mechanisms underlying strategies I and II represent valuable targets for plant synthetic biology, as reprogramming their expression and spatiotemporal activity could decouple iron uptake from native regulation (e.g., iron limitation) to produce iron-deficiency-tolerant, hyperaccumulating crops.

**Figure 3:**
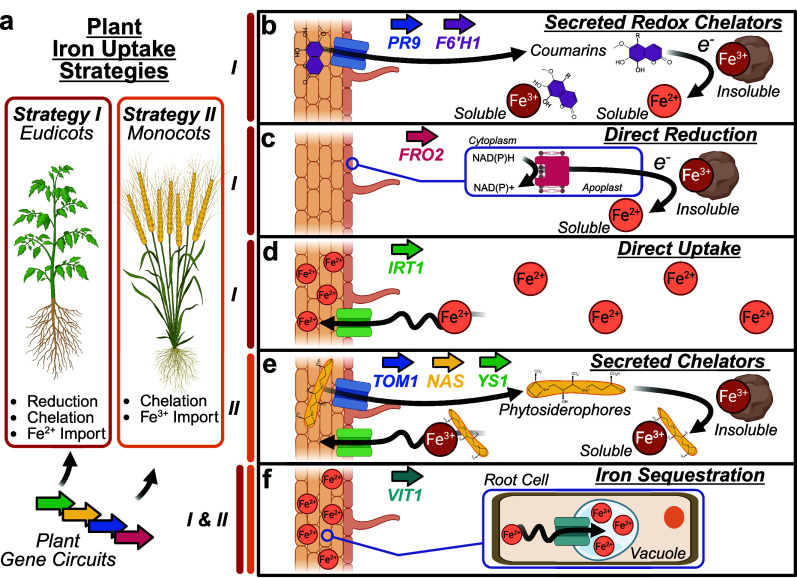
Synthetic biology targets for enhancing plant iron uptake. (a) Strategy I and II iron acquisition pathways can be engineered using synthetic gene circuits. (b) Strategy I plants secrete coumarins that act as both chelators and reductants for solubilizing iron. (c) Membrane-bound reductases, like FRO2, are used by strategy I plants to reductively solubilize iron. (d) Strategy I plants uptake Fe^2+^ via the IRT1 transporter. (e) Strategy II plants secrete phytosiderophores (e.g., mugineic acid) via the TOM1 transporter, and reuptake Fe^3+^ chelates via the YS1 family transporters. (f) Vacuolar transporters, including VIT1 sequester iron within organelles to increase overall accumulation.

**Table 2:** Representative Plant Engineering Targets for Enhancing Rhizosphere Iron Mobilization, Extraction from Soil, and Sequestration within Plant Tissues

**molecule or protein**	**midpoint potential (mV vs SHE)**	**Fe** ^**3+**^ **dissociation constant**, ***K***_**d**_ **(M)**	**relevant taxa**	**references**
common redox couples in plants				
Fe^3+^/Fe^2+^	+770		universal	
NADP^+^/ NADPH	–320		universal	^107^
secreted redox chelators (coumarins)				
sideretin	+228, +503	7.9 × 10^–18^	*A. thaliana,* Eudicots	^73^,^108^,^109^
fraxetin	+492, +803	not reported	*A. thaliana,* Eudicots	^73^,^108^
esculetin	+603	not reported	*A. thaliana,* Eudicots	^108^
scopoletin	+905	not reported	*A. thaliana,* Eudicots	^108^
phytosiderophores				
mugineic acid		2 × 10^–18^	*Zea mays*, *Oryza sativa*, Poaceae	^110^
2′-deoxymugineic acid		4 × 10^–19^	*Z. mays*, *O. sativa*, Poaceae	^110^
membrane-bound or extracellular reductases				
FRO2	not reported		*A. thaliana*, Eudicots	^26^
cytochrome b561 (e.g., AIR12, CRR, and HYP1)	–29 to +190		*A. thaliana*, Eudicots	^111^

Coumarins are key root exudates of strategy I plants, which mobilize rhizosphere iron through both chelation and reduction mechanisms (Figure 3b). Major coumarins, including sideretin and fraxetin, are synthesized from the central precursor scopoletin, making the scopoletin biosynthetic gene *F6′H1* a promising metabolic engineering target to boost overall coumarin production.^73,74^ Given the chemical diversity of coumarins and their varying effects on iron extraction, exudate profiles could be tuned via circuits expressing specific coumarin synthases, such as scopoletin hydroxylase S8H for fraxetin or cytochrome P450 CYP82C4 for sideretin.^73^ While coumarin export circuits have yet to be explored, expression of characterized transporters like PDR9 could enhance exudation rates and increase coumarin abundance in the rhizosphere.^75,76^ As optimization of coumarin exudation will likely require coexpression of multiple genes encoding biosynthesis and transport, plant-functional polycistronic units could be built by fusing genes in tandem and linking them with self-cleaving 2A or IRES peptide sequences.^77^

While many root exudates act as diffusible reductants and iron solubilizers, strategy I plants employ root cell membrane-bound oxidoreductases to directly reduce Fe^3+^ to Fe^2+^, enhancing iron mobilization and uptake (Figure 3c). The best characterized example of this is the FRO family of reductases,^78^ particularly FRO2,^79^ which couples cytoplasmic NAD­(P)H oxidation to apoplastic Fe^3+^ reduction. *FRO* genes are differentially expressed across plant tissues and tightly regulated in response to various environmental triggers.^80,81^ Under iron deficiency, *FRO2* is confined to root epidermal membranes while *FRO3* is expressed in the root apical meristem.^82,83^ As *FRO2* overexpression has been shown to improve plant growth on low iron,^26^ circuits that broaden *FRO2* expression beyond epidermal layers or facilitate functional transfer into strategy II plants might yield improved iron uptake by crops. Other plant reductases also represent promising targets for the reduction of apoplastic iron. Although additional membrane-bound oxidoreductases from the cytochrome b561, heme- and sugar-binding DOMON, and CYBDOM (cytochrome b561–DOMON fusions) families have been identified, their substrate range, physiological roles, and potential for circuit-based iron mobilization remain largely uncharacterized. For instance, the NADPH/quinone oxidoreductase NQR transfers electrons to the apoplast-facing DOMON-containing *b*-type cytochrome AIR12, which leads to the modulation of apoplastic ROS levels.^84^ CYBDOM reductases CRR and HYP1 couple Fe^3+^ reduction to ascorbate oxidation, thereby influencing apoplastic iron deposition.^83,85^ Collectively, these genes represent unique redox actuators for plant synthetic biology, and their systematic characterization via gene circuits could both uncover fundamental biology and improve iron availability for crops.

Specialized transporters of strategy I plants mediate the uptake of solubilized iron from soil and its mobilization within plant tissues, representing another valuable class of plant synthetic biology parts (Figure 3d). The ZIP transporter family consists of proteins that facilitate preferential or iron-specific transport, with IRT1 being the most extensively characterized and genetically tractable transporter in model plants. IRT1 is primarily expressed in roots and flowering tissues under iron-deplete conditions, and its knockout leads to severely stunted growth and lethality.^86,87^ However, constitutive overexpression can lead to excess iron accumulation, which promotes Fe­(II)-mediated reactions with oxygen and generates hydroxyl radicals.^88^ These reactive oxygen species (ROS) are highly toxic, damaging cell membranes and disrupting photosynthesis, often resulting in leaf chlorosis and cell death.^88,89^ One solution to mitigate this is the use of synthetic circuits that spatially and temporally regulate IRT1 to balance iron uptake and toxicity, which is particularly important for iron deficiency-susceptible crops like soybean.^90^ IRT1 has been tested with a range of tissue-specific constructs beyond its native epidermal and cortical root cell localization, using a panel of root-specific *A. thaliana* promoters.^91^ Although all single promoter–*IRT1* fusions failed to restore growth in the *irt1*-1 knockout background, simultaneous use of two non-native cell-type-specific promoter–*IRT1* fusions (*proEXP7*–trichoblast and *proSUC2*–phloem companion cell) rescued wild-type growth under iron deficiency. These results suggest that physiological design rules must be considered when expanding transporter expression beyond native territories and that Boolean logic circuits^92^ could fine-tune cell-type specificity with smaller genetic footprints and enable inducible expression in response to environmental cues.

Among the strategy II phytosiderophores, mugineic acid (MA) is the predominant form produced by grasses under iron stress^72,93^ and is conserved across maize, rice, wheat, and other Poaceae^94^ (Figure 3e). Roots synthesize this tricarboxylic acid chelator from methionine-derived pathways,^94,95^ export it via the TOM1 transporter,^96^ and reimport Fe^3+^-MA chelates through the YS1/YSL15 transporter family.^97,98^ Genetic circuits that modulate MA biosynthesis and transport could be broadly deployed across food-relevant monocots and potentially introduced into strategy I species, as demonstrated in petunia.^99^ For example, constitutive overexpression of the barley nicotianamine synthase gene (*HvNAS1*), a controller of MA precursor biosynthesis, via the 35S promoter led to a 3-fold increase in the iron concentration in rice.^100^ This suggests that *HvNAS1* and related phytosiderophore synthase genes^101^ could serve as metabolic engineering targets for iron biofortification. A major limitation to phytosiderophore efficacy in monocots is the microbial degradation of MA in soil.^93,102^ Notably, a synthetic phytosiderophore with a similar structure to MA, called PDMA, retains strong Fe^3+^ chelation yet resists microbial metabolization.^93^ Engineering phytosiderophore biosynthetic pathways to produce PDMA or other structural analogues, such as by expressing *NAS* orthologues,^100^ could represent a promising strategy to enhance crop iron acquisition.

While we have mainly focused on synthetic biology approaches to improve the limiting step of iron extraction from soil, gene circuits that increase iron storage within plant tissues could further fortify food crops. A relevant genetic target in both strategy I and II plants is the vacuolar iron transporter VIT1, which plays a critical role in transport and sequestration of iron into vacuoles during seed development^103^ (Figure 3f). Overexpression of VIT1 has led to substantial increases in iron levels for food crops like cassava,^104^ where it was targeted to storage roots via the *Solanum tuberosum* type I patatin promoter. These simple manipulations suggest that more complex circuitry could further increase iron storage to parts of the plant inedible to humans but may serve as feed for animals (e.g., maize silage). Alternatively, the use of field-inducible promoters (e.g., pDEX^105^) may facilitate appropriately timed expression of *VIT1* during crop growth to maximize iron bioavailability for harvest.^103,106^ By using inducible promoters that target different plant tissues or developmental stages, iron acquisition and translocation could be decoupled from native regulation and systematically optimized to hyperaccumulate tissue iron while mitigating free iron-driven ROS generation.

## Future Outlook

The centrality of iron to both plant and human health underscores the vast potential for using plant and rhizobacterial synthetic biology to optimize food crop biofortification. Although regulatory approval for the release of engineered PGPB remains a significant hurdle,^112^ the rapid design–build–test–learn cycle of rhizobacteria equipped with genetic circuits that enhance iron solubilization and uptake is likely to enable faster field deployment than comparable strategies in plants. Certain strain engineering approaches with fewer regulatory barriers (e.g., intrageneric genome modification, adaptive evolution, and random mutagenesis) could further fast-track deployment by enhancing native iron-mobilizing bacteria to improve rhizosphere colonization^44^ or constitutively express iron mobilization genes (e.g., oxygen-insensitive *mtrCAB* activity^113^). Beyond the well-characterized genetic and metabolic targets that we highlight, recently developed synthetic biology tools for prototyping complex metabolic pathways could accelerate the discovery of iron homeostasis actuators, such as novel siderophores,^114^ that are likely abundant across poorly characterized rhizosphere metagenomes.^29,115,116^ Genetic circuits incorporating sensor- and logic gate-driven programming could further optimize iron mobilization alongside other PGPB traits (e.g., nitrogen fixation^28^), enabling multimodal bioinoculants with dynamic pathway activation that reduces metabolic burden through conditional expression in response to plant and environmental cues.

While we mainly consider the direct effects of bacterial bioinoculants on plant iron acquisition, we also note that other soil microbes may both influence and respond to altered rhizosphere iron mobilization. For example, boosting PGPB-mediated Fe^3+^ reduction may augment the activity of iron oxidizing bacteria that couple the oxidation of resulting Fe^2+^ to energy conservation.^117^ Although the ecology of these microbes in rhizosphere soils remains unclear, iron oxidizers are known to secrete metabolites that stabilize Fe^2+^ against abiotic oxidation,^118^ and increased activity of these strains may act synergistically with iron-reducing PGPB to enhance the availability of soluble iron for plant uptake. Similarly, arbuscular mycorrhizal fungi play key roles in plant iron acquisition, and the effects of augmented iron solubilization by PGPB may be enhanced in the presence of these fungi.^119^ Beyond understanding the impact of iron-mobilizing PGPB on other soil microorganisms, these currently genetically intractable microbes may also serve as a chassis for engineering iron mobilization as new genetic tools become available. Finally, future efforts should also optimize the persistence of engineered strains within native microbial communities, where competition can limit the target strain growth and performance. Engineering strain use of rhizosphere carbon,^120^ assimilation of inorganic nutrients such as iron,^121^ and the mode of bioinoculation (e.g., seed coating^122^) will all influence the long-term stability of strains within complex microbiomes and ultimately the effectiveness of engineered iron mobilization functions.

Microbes remain the fastest organisms to engineer, but next-generation plant transformation protocols are poised to accelerate genetic circuit design for iron mobilization and sequestration. Emerging techniques such as nanoparticle-mediated gene delivery^123^ or the tissue culture-free cut–dip–budding method^124^ could facilitate engineering these functions across both model and nonmodel plants. Moreover, insights from well-characterized plant iron homeostasis pathways could inform their integration into synthetic circuit architectures that leverage machine learning- or AI-guided cell-type engineering^125^ or protein engineering approaches (e.g., altering transporter specificity^126^). These tools would expand traits controllable via plant synthetic biology, which to date have focused on altering plant development^92^ and biochemical compositions.^127^ They may also enable synergistic plant–microbe engineering to enhance plant iron acquisition, taking advantage of complementary traits in each organism. For instance, as plants can uptake bacterial siderophore–iron complexes through mechanisms that remain largely unknown,^128^ identifying the underlying plant genes could open the door to transkingdom engineering of plants and bioinoculants. Because conventional plant synthetic biology outputs mainly affect intracellular processes, iron-controlling plant genes also offer a distinct class of actuators capable of modulating both plant tissues and plant–environment interactions. For instance, precise regulation of plant–microbe–metal interactions could enable applications in microbiome engineering,^121^ phytoremediation,^129^ and biomining of critical minerals.^130^ Such circuits may include overexpression of rhizosphere pollutant detoxification pathways, such as the bacterial *ars* operon for arsenic tolerance,^131^ or plant transporter proteins for rare earth element sequestration (e.g., *NREET*).^130^ With growing interest in microbe–iron interactions for biomanufacturing and biomaterials,^132^ plant–microbe–iron synthetic biology is strongly positioned to improve both yields and nutritional quality in food systems.
